# Investigation of household contacts of pulmonary tuberculosis patients increases case detection in Mwanza City, Tanzania

**DOI:** 10.1186/s12879-018-3036-6

**Published:** 2018-03-06

**Authors:** Medard Beyanga, Benson R. Kidenya, Lisa Gerwing-Adima, Eleanor Ochodo, Stephen E. Mshana, Christa Kasang

**Affiliations:** 1 Bugando Medical Centre, Department of Clinical Laboratory Services, Mwanza, Tanzania; 2 Department of Biochemistry and Molecular Biology, Weill Bugando School of Medicine, Mwanza, Tanzania; 3 Centre for Evidence-based Health Care, Stellenbosch University Cape Town, Cape Town, South Africa; 4 Department of Microbiology, Weill Bugando School of Medicine, Mwanza, Tanzania; 5 Medical Mission Institute, Wuerzburg, Germany

**Keywords:** Tuberculosis, Bacteriologically-confirmed pulmonary tuberculosis, Household TB contacts, Active TB case finding, Mwanza, Tanzania

## Abstract

**Background:**

Tuberculosis (TB) contact tracing is a key strategy for containing TB and provides addition to the passive case finding approach. However, this practice has not been implemented in Tanzania, where there is unacceptably high treatment gap of 62.1% between cases estimated and cases detected. Therefore calls for more aggressive case finding for TB to close this gap. We aimed to determine the magnitude and predictors of bacteriologically-confirmed pulmonary TB among household contacts of bacteriologically-confirmed pulmonary TB index cases in the city of Mwanza, Tanzania.

**Methods:**

This study was carried out from August to December 2016 in Mwanza city at the TB outpatient clinics of Tertiary Hospital of the Bugando Medical Centre, Sekou-Toure Regional Hospital, and Nyamagana District Hospital. Bacteriologically-confirmed TB index cases diagnosed between May and July 2016 were identified from the laboratory registers book. Contacts were traced by home visits by study TB nurses, and data were collected using a standardized TB screening questionnaire. To detect the bacterioriologically-confirmed pulmonary TB, two sputum samples per household contact were collected under supervision for all household contacts following standard operating procedures. Samples were transported to the Bugando Medical Centre TB laboratory for investigation for TB using fluorescent smear microscopy, GeneXpert MTB/RIF and Löwenstein–Jensen (LJ) culture. Logistic regression was used to determine predictors of bacteriologically-confirmed pulmonary TB among household contacts.

**Results:**

During the study period, 456 household contacts from 93 TB index cases were identified. Among these 456 household contacts, 13 (2.9%) were GeneXpert MTB/RIF positive, 18 (3.9%) were MTB-culture positive and four (0.9%) were AFB-smear positive. Overall, 29 (6.4%) of contacts had bacteriologically-confirmed pulmonary TB. Predictors of bacteriologically-confirmed pulmonary TB among household contacts were7being married (Odds ratio [OR], 3.3; 95% confidence interval [CI], 1.4–8.0; *p* = 0.012) and consuming less than three meals a day (OR, 3.7; 95% CI, 1.6–8.7; *p* = 0.009).

**Conclusions:**

Our data suggest that in Mwanza, Tanzania, seven in 100 contacts living in the same house with a TB patient develop bacteriologically-confirmed pulmonary TB. These results therefore underscore the need to implement routine TB contact tracing to control tuberculosis in high TB burden countries such as Tanzania.

## Background

Tuberculosis (TB) is a deadly disease primarily transmitted through airborne droplets, particularly in populations that share air with infected individuals [1]. In TB high-burden countries, the majority of cases are diagnosed when patients seek health care at health-care facilities (“passive case finding”) [2, 3]. Active screening of individuals in contact with TB-infected cases is a key component in containing the disease in low-incidence countries. However, this practice has not been implemented in most high-burden countries [4].

The 2016 World Health Organization report on TB ranked Tanzania among the 22 countries with the highest tuberculosis burden worldwide [5]. To control TB, healthcare systems will need to detect more cases of TB at an earlier stage of the illness [6]. However, in 2016 WHO estimates high number of cases not reported and treated in high burden countries [5]. In Tanzania, WHO estimates 164,000 people had TB disease, of these, only 62,180 (37.9%) cases were notified [5]. This demonstrates a huge treatment gap of 62.1% of all people with TB in Tanzania were not reported and treated in Tanzania. This treatment gap is unacceptably high and calls for more aggressive case finding for TB (household contacts via active case findings) to close this gap. Active case finding by TB contacts tracing provides a promising addition to the passive case finding approach [7], as it a very effective method of increasing case detection rates [8]. However, this practice has not been implemented in Tanzania. A systematic review and meta-analysis of 41 studies showed that screening household contacts in middle- and low-income countries increased new case findings by 4.5% [1]. It is imperative to prioritize active case finding, as it leads to early diagnosis and shortens exposure of cases within the community [9–11].

Analysis of studies has indicated that, where the national prevalence of TB reaches 100 cases per 100,000 people, the active case-finding technique detected one case among 100 tested individuals [12]. Two studies in Tanzania showed having less education and living far from testing facilities to be factors associated with delayed case detection [13, 14]. Studies in three countries in West Africa have shown that the host and environmental factors, predicting pulmonary TB were male sex, HIV infection, smoking, history of asthma, family history of TB, marital status, adult crowding, and renting the house [15]. Studies outside Africa have shown the predictors of bacteriologically-confirmed pulmonary TB among household contacts include: male sex, low body weight, alcoholism, glucocorticoid therapy, and diabetes [16].

The WHO strongly recommends the practice of systematic screening of active TB household contact, people living with HIV, mining communities, workers exposed to silica, and imprisoned people. This practice is encouraged in general communities where prevalence is ≥100 per 100,000 people [17]. Active case finding is important in diagnosing new TB cases, which offers the opportunity to treat infected patients at an earlier stage, before signs and symptoms develop, especially in HIV endemic areas as HIV fuels TB transmission [18].

Therefore, this study aimed to determine bacteriologically-confirmed pulmonary TB among household contacts of bacteriologically-confirmed pulmonary TB index cases in the city of Mwanza, Tanzania. It also determined predictors of bacteriologically-confirmed pulmonary TB among household contacts. A systemic review and meta-analysis of 41 studies showed that screening household contacts increased new case findings by 4.5% [1]. Therefore, we hypothesized that there is ≥5% of new bacteriologically-confirmed pulmonary TB cases among these index cases in the city of Mwanza.

## Methods

From August to December 2016, a retrospective study was done on newly bacteriologically confirmed TB index cases between May and July 2016 at TB outpatient clinics at Bugando Medical Centre (BMC) Tertiary Hospital, Sekou-Toure Regional Hospital, and Nyamagana District Hospital in the city of Mwanza. A retrospective chart review from the laboratory registers book was done to identify new bacteriologically confirmed TB index cases. These index cases received home visits by the study TB nurses and household contacts were screened for TB using a standardized TB screening questionnaire.

Demographic information and address and phone numbers of the index cases were extracted from registry books. Before visits, a call was made to the index case to inform of the visit, arrange the day and suitable time, and discuss the possibility of meeting all house inhabitants. Calls were made using the telephone number extracted from the TB laboratory registers. The study team visited all cases who accepted.

Index case household contacts who were willing to participate were asked to sign giving their consent, and were requested to provide a spot sputum sample regardless of TB signs or symptoms. Those who provided a sample were left with a sputum container for depositing the morning specimen.

Special arrangements were made to accommodate contacts that were unavailable during the first visit. We optimized our effort to collect sputum samples from children by instructing and supervising them in producing sputum. Contacts with clinical signs, especially children who were unable to produce sputum, were referred to the nearby TB clinic and managed in accordance with the national TB guidelines.

The household contacts who were able to produce the sputum sample were enrolled into this study while those who were not able to produce sputum were excluded. The study did not collect and report data on those excluded based on refusal of participation, could not be reached, or could not produce sputum. At the time of home visits, the study TB nurses administered questionnaires to only those who provided spot sputum samples, to obtain the socio-demographic, baseline information, and predictors of bacteriologically-confirmed pulmonary TB. The predictors investigated were symptoms of pulmonary TB (cough, fever, weight loss, excessive night sweats and hemoptysis), current smoking, house size, distance to health facility, number of people living in the house and number of meals taken per day. Later, the house parameters were measured using a tape measure.

### Study definitions

Bacteriologically-confirmed pulmonary TB was defined as the individual having at least one positive result from AFB smear, GeneXpert MTB/RIF (Cepheid, Sunnyvale, CA, USA) or LJ culture. We used a definition of a household contact as defined elsewhere but with a stringent criteria on time. In this context a household contact was defined as a person who had been in close and frequent contact by living in the same house with a TB index case for at least 3 days in between 3 weeks before diagnosis and 1 week after diagnosis and commencement of treatment [19].

### Sample collection and laboratory analysis

Contacts were instructed and supervised to collect one spot sample and one morning sputum sample generated from a deep cough. The samples were analyzed by GeneXpert MTB/RIF. In cases where this was not sufficient, the second sample was used. Additionally, two culture slopes and two smears were made from each sample. We performed all three tests (GeneXpert MTB/RIF, LJ culture and fluorescent smear microscopy) for all 456 contacts at BMC TB laboratory. Routine laboratory standard operating procedures were used to analyze the samples. Bacterial culture was performed using Löwenstein–Jensen (LJ) medium base. All standard operating procedures were validated using known samples and the validation reports were documented for traceability. Staff who analyzed samples were trained and assessed for competency to ensure procedures were properly followed. Equipment for analyzing samples was validated and maintained in accordance with the respective manufacturer’s instructions*.* TB control strains were cultured against samples to control the culturing process.

### Data management and statistical analysis

Laboratory personnel collected laboratory data, while a TB nurse collected clinical data about the symptoms for TB. All data generated from home visit and laboratory work were first collected in hard copy, except for GeneXpert MTB/RIF results, which were generated by the machine. Data were then double entered into Epi Data version 3.1 (The Epi Data Association, Odense, Denmark). Accessing of data was limited to authorized personnel and amendment of records was not allowed. The researcher maintained all participants’ data in a securely locked cupboard. Information on participants’ positive TB diagnoses was forwarded to the TB coordinator to assist with patient treatment.

Data were then imported into STATA 13 (Stata Corp LLC, College Station, TX, USA) for management and analysis. We reported the median with interquartile range (IQR) for continuous data and simple frequencies (numbers), and percentages (proportions) for categorical data. The main outcome was bacteriologically-confirmed pulmonary TB detected by contact tracing. The factors assessed were household contact’s relationship with the TB index cases, TB symptoms, level of education, house size, smoking, age, sex, marital status, number of meals per day, number of family members, and distance to the nearest health center. We employed univariate followed by multivariate logistic regression models to determine factors associated with bacteriologically-confirmed pulmonary TB among contacts. Multivariate logistic regression was conducted on factors that were statistically significant in univariate regression. Odds ratio (OR) with its 95% confidence interval (CI) was computed.

### Informed consent and ethical clearance

Study participants’ consent was obtained after identification of TB-positive cases from a laboratory register in Mwanza. The participants were given clear explanation about the project and how they would participate, as well as the benefits. A consent form was signed upon agreement to participate. Consent forms were translated in the Swahili National language. For participants who were unable to read, their consent were witnessed by explaining the purpose of the study and its benefits. The study team clearly explained that participation into the study was absolutely voluntary and participants were allowed to leave the study at any time. For children below 18 years, informed consent was sought from parents or guardians as per Tanzania medical research regulations.

The study was approved by the ethics committees of both the Joint Catholic University of Health and Allied Science/Bugando Medical Centre (Mwanza, Tanzania) and Stellenbosch University (Cape Town, South Africa).

### Referral of contacts with bacteriologically-confirmed pulmonary TB

Identified TB-positive contacts from any of our testing methods were referred to the nearby TB clinic receive free TB treatment. Contacts especially children who were not able to produce sputum but had presented with signs and symptoms of TB were referred to nearby TB clinic for further investigations.

## Results

### Description of participant characteristics

Between May and July, a total of 93 TB index cases were identified from laboratory registers. Upon following of these cases, 456 household contacts met eligibility criteria and were enrolled into the study. The median age for the household contacts was 22 (IQR, 15–37) years. A slight majority of participants were female 57.0% (260/456), and 55.5% (253/456) were married. The most common contact relationships with cases were siblings at 41% (187/456), followed by parents at 24.6% (112/456). More than half of the contacts, 59.2% (270/456), reported they had three or more meals a day. There were 4.4% (20/456) household contacts who were habitual smokers (Table 1).

**Table 1:** Characteristics of 456 household contacts in Mwanza, Tanzania

Contacts character	Number (n)	Percent (%)
Age in years		
< 5	6	1.3
5–14	66	14.5
15–19	45	9.9
20–24	60	13.2
25–44	182	39.9
45–64	85	18.6
> 65	12	2.6
Sex		
Male	196	43.0
Female	260	57.2
Education level completed		
Illiterate	76	16.7
Primary	285	62.5
Secondary	88	19.3
Tertiary	7	1.5
Marital Status		
Married	253	55.5
Single^a^	203	44.5
Family members		
< 5	37	8.1
5–9	185	40.6
10–14	183	40.1
15–20	51	11.2
Relationship		
Child	79	17.3
Siblings	187	41.0
Spouse	24	5.3
Uncle-Aunt	24	5.3
Parent	112	24.6
Grand parent	30	6.6
Number of meals		
< 3	186	40.8
≥ 3	270	59.2
Distance to Health facility		
< 1Km	92	20.2
1–4.9 Km	334	73.2
5–10 Km	30	6.6
Smoking		
No	436	95.6
Yes	20	4.4
Cough		
No	16	3.5
Yes	440	96.5
Fever		
No	342	75.0
Yes	114	25.0
Cough blood		
No	445	97.6
Yes	11	2.4
Weight loss		
No	417	9.5
Yes	39	8.6
Night sweat		
No	299	65.6
Yes	157	34.4

^a^Widowed and divorced were handled as single

**Fig. 1:**
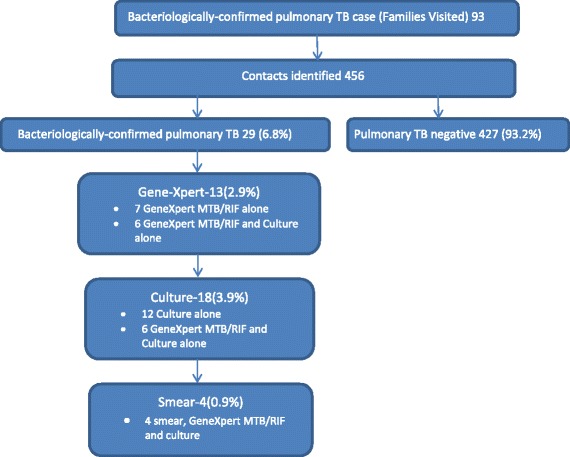
Pulmonary TB case finding results summary**.** *We regret that we did not quantify the numbers of unreachable, refusing and/or not producing sputum besides their remarkable importance in this study

Among the 456 household contacts, 13 (2.9%) were positive for GeneXpert MTB/RIF, 18 (3.9%) were *M.tb-*culture positive and and four (0.9%) were AFB-smear positive. Four were positive for all three tests. Six were only positive for both GeneXpert MTB/RIF and culture. Seven contacts only GeneXpert MTB/RIF positive, whereas 12 were only positive for the culture. None were positive for only the smear, smear and culture, and smear and GeneXpert MTB/RIF. There were 10 (2.1%) samples that had invalid results on GeneXpert MTB/RIF and 16 (3.5%) contaminated in the culture. Overall, 29 (6.4%) were bacteriologically diagnosed TB cases (Fig. 1).

### Predictors of bacteriologically-confirmed pulmonary TB among household contacts

Following multivariate logistic regression analysis, the independent predictors of bacteriologically-confirmed pulmonary TB among household contacts were: being married (OR, 3.3; 95% CI, 1.4–8.0; *p* = 0.012) and consuming less than three meals a day (OR, 3.7; 95% CI, 1.6–8.7; *p* = 0.009). (Table 2).

**Table 2:** Factors associated with bacteriologically-confirmed pulmonary tuberculosis among 456 household contacts

Contact characteristics	Tuberculosis		Univariate		Multivariate	
	Yes n (%)	No n (%)	OR [95% CI]	*p*-value	OR [95% CI]	*p*-value
Age in years						
< 5	1 (16.7)	5 (83.3)	1.0			
5–14	3 (4.6)	63 95.4)	0.2 [0.02 – 2.7]	0.249	-	-
15–19	2 (4.4)	43 (95.6)	0.2 [0.02 – 3.0]	0.269	-	-
20–24	6 (10.0)	54 (90.0)	0.6 [0.06 – 5.6]	0.617	-	-
25–44	10 (5.5)	172 (94.5)	0.3 [0.03 – 2.7]	0.280	-	-
45–64	7 (8.2)	78 (91.8)	0.4 [0.05 – 4.4]	0.491	-	-
> 65	0 (0.0)	12 (100.0)	-	-	-	-
Sex						
Male	20 (7.7)	240 (92.3)	1.0			
Female	9 (4.6)	187 (95.4)	0.6 [0.3 - 1.3]	0.184	-	-
Education						
Illiterate	7 (9.2)	69 (90.8)	1.0			
Primary	18 (6.3)	267 (93.7)	0.7 [0.3 – 1.7]	0.380	-	-
Secondary/Tertiary	4 (4.2)	91 (95.8)	0.4 [0.1 – 1.5]	0.196	-	-
Marital Status						
Single	7 (3.5)	196 (96.6)	1.0			
Married	22 (8.7)	231 (91.3)	3.3 [1.1 – 6.4]	0.027	3.3[1.4 – 8.0]	0.012
Relationship						
Child	7 (8.9)	72 (91.1)	1.0			
Siblings	10 (5.3)	177 (94.7)	0.6 [0.2 – 1.6]	0.289	-	-
Spouse	2 (8.3)	22 (91.7)	0.9 [0.2 – 4.9]	0.936	-	-
Uncle-Aunt	1 (4.2)	23 (95.8)	0.4 [0.1 – 3.8]	0.463	-	-
Parent/Grand parent	9 (6.3)	133 (93.7)	0.7 [0.3 – 2.0]	0.490	-	-
Number of people per 5 square metres						
< 5	4 (10.8)	33 (89.2)	1.0			
5–9	14 (7.6)	171 (92.4)	0.7 [0.2 – 2.2]	0.512	-	-
10–14	10 (5.5)	173 (94.5)	0.5 [0.1 – 1.6]	0.233	-	-
15–20	1 (2.0)	50 (98.2)	0.2 [0.02 – 1.5]	0.114	-	-
Number of meals per day						
> 3 meals	12 (4.5)	258 (96.5)	1.0			
≤ 3 meals	17 (9.1)	169 (90.9)	2.1 [1.1 – 4.60]	0.048	3.7 [1.6 – 8.7]	0.009
Distance to health facility						
< 1 Km	3 (3.3)	89 (96.7)	1.0			
1–4.9 Km	23 (6.9)	311 (93.1)	2.2 [0.6 – 7.5]	0.209	-	-
5–10 Km	3 (10.0)	27 (90.0)	3.2 [0.6 – 17.3]	0.158	-	-

## Discussion

Using active case finding among household contacts of bacteriologically-confirmed adult pulmonary TB cases, we found that 6.4% of household contacts who produced a sputum sample also had bacteriologically confirmed pulmonary TB in Mwanza, Tanzania. These results mean the active case-finding technique provides an increased TB detection rate about 20 times higher than the 306 per 100,000 people detection rate achieved by passive diagnosis [5].

Our results provided a high yield compared with Uganda, which was 3.5% [9], and 4.5% from a systematic review involving 41 case-finding studies that was conducted to synthesize evidence of implementing active case finding as an effective approach for raising the TB-detection rate [1]. The results from the present study were consistent with active case findings in urban slums in southeastern Nigeria, which reported 6.4% of TB cases undiagnosed [20]. Our results therefore reinforce the WHO recommendation to implement TB case-finding targeting areas at high risk of TB (e.g., mines and prisons).

Our results found a statistically significant association between bacteriologically-confirmed pulmonary TB and taking less than three per day. This could be explained by the fact that people with good nutrition had improved immunity [21, 22]. Nutrition plays a major role in the management of both acute and chronic diseases, in terms of body’s response to the pathogenic organism [21–23]. An array of nutrients like macro- and micro-nutrients (vitamins, minerals, and trace elements) are associated with boosting the host’s immune responses against intracellular pathogens including *Mycobacterium tuberculosis* [23]. These nutrients have an immuno-modulatory effects in controlling the infection and inflammation process. Hence nutritional deficiency of any form may greatly increases an individual’s susceptibility to progression of infection to disease [23].

The majority of contacts with bacteriologically-confirmed pulmonary TB were married. This high proportion may be due to the fact that married people share a bedroom with their spouses, and are in closer contact with other people as well such as their children, friends, etc. than a single person. Thus, perhaps that closer contact to spouse, and to children, friends, etc., predisposes them to an infectious contact than any other person in the house [24].

We anticipated the number of people staying in houses per square meter of floor space (crowding), relationship of contacts with TB cases, and education level could be associated with TB among household contacts. Our results on risk factors differed from in a multicenter study done in West Africa that looked at risk factors for TB; that study found an association between adult crowding and renting of houses [15]. Our results show no statistically significant association between the mentioned factors.

The limitations to this study were we did not follow up house-hold contacts for some times and did not induce sputum to those unable to produce it especially children and also did not diagnose clinical pulmonary tuberculosis and extra-pulmonary TB. If these could be done the yield of active case finding could be higher. There could the possibility of bias in contact tracing and confounding, and the therefore the sample size could not be the representative for the city of Mwanza. Examining HIV was outside the scope of this study, but remains critically important for any intervention targeting TB.

## Conclusions

Our data suggest that seven in 100 TB household contacts had bacteriologically-confirmed pulmonary TB. This figure underscores the need for implementation of routine contact tracing to control TB in developing countries. Further studies with follow up component are warranted on clinical tuberculosis, sputum induction, especially on children and those who can not produce sputum. Furthermore, based on this association, programs targeting diet should be integrated into TB services.

## Abbreviations

HIV: Human Immunodeficiency Virus
LJ: Löwenstein–Jensen
MTB/RIF: Mycobacterium tuberculosis/Rifampicin
TB: Tuberculosis
WHO: World Health Organization
